# Influence of visual control on the quality of graphic gesture in children with handwriting disorders

**DOI:** 10.1038/s41598-021-02969-7

**Published:** 2021-12-07

**Authors:** Clémence Lopez, Laurence Vaivre-Douret

**Affiliations:** 1 Faculty of Society and Humanity, Department of Psychology, Université de Paris, Paris, France; 2 National Institute of Health and Medical Research (INSERM UMR 1018-CESP), Paris-Saclay, UVSQ, Villejuif and Necker-Enfants Malades University Hospital, Carré Necker Porte N4, 149, rue de Sèvres, 75015 Paris, France; 3 Faculty of Health, Department of Medicine, Université de Paris, Paris, France; 4 Institut Universitaire de France (IUF), Paris, France; 5 Necker-Enfants Malades University Hospital, AP-HP.Centre, Paris, France; 6 Department of Paediatric Endocrinology, Imagine Institute, Necker-Enfants Malades University Hospital, Paris, France

**Keywords:** Cognitive neuroscience, Cognitive control

## Abstract

Handwriting disorders (HD) are considered one of the major public health problems among school-aged children worldwide with significant interference on academic performances. The current study hypothesized that HD could be partly explained by a deficit in sensory feedback processing during handwriting. To explore this hypothesis, we have analyzed the effect of vision suppression on postural-gestural and on spatial/temporal/kinematic organization of drawing during an early pre-scriptural loop task with a digital pen, under two conditions: eyes open and eyes closed. Data collected from 35 children with HD were compared to data collected from typical children (typical group) from primary schools. The HD group showed significantly poorer postural control and an improvement on the spatial/temporal/kinematic organization of drawings when they closed their eyes compared to eyes opened. While in the typical group, postural-gestural organization became significantly more mature but there was no significant influence found on spatial/temporal/kinematic parameters of the loops. Thus, handwriting disorders could be explained by both proprioceptive/kinesthetic feedback disabilities and a disruptive effect of the visual control on the quality of the pre-scriptural drawings among these children who have kinesthetic memory and visuospatial disabilities. The ability of directing the strokes would remain dependent on sensory feedbacks, themselves insufficiently efficient, which would lead to difficulties in reaching a proactive control of handwriting. This current research is a liable contribution to enhance clinical practice, useful in clinical decision-making processes for handwriting disorders remediation.

## Introduction

Handwriting disorders are considered one of the major public health problems among school-aged children worldwide, with a prevalence rate of handwriting disorders in school-aged children ranging from 6 to 33%^1^. These disorders significantly interfere with academic performance and are often associated with learning difficulties, for example in spelling and story composition^2^.

During writing, the eyes guide the hand to write the letters, to arrange them in the writing space and to return (back) to the writing line. Visual control allows to move from spontaneous drawing traces, guided by the kinesthetic aspects of the gesture, to controlled and directed sequences of strokes. Several authors^3,4^ explain that visual control becomes more important to compensate for the decrease in kinesthetic feedback. This interdependence of visual and kinesthetic controls of the graphomotor gesture has been supported by the knowledge developed on the integration of the motor programs of letters during the learning of writing^5–8^. This integration moment of motor programs corresponds to transition from control by sensory feedback (mainly visual but also proprioceptive, predominant at the beginning of learning) to a proactive control of handwriting (development of an internal representation of movement less dependent on sensory feedback). When learning cursive writing, the child learns to form the letters and to link them together around the age of 6. At this time, the child does not perceive the letter as a whole and produces it by the juxtaposing small segments. The trajectory of the strokes is imprecise and the letter is segmented, which manifests itself by a dented aspect (stroke by stroke) of the letter. This mode of production is dependent on feedback control movement strategies in which the child relies heavily on visual feedback to control stroke trajectory. At this point, the child regularly returns to the model. Visual feedback provides information about the spatial characteristics of the letters, while proprioceptive/kinesthetic feedback provides information about the position and coordination of the segments and joints of the writing arm and the pressure exerted on the pen^9^. At around 8–9 years of age, the child begins to integrate the shape of the letters into long-term memory and thus to automate the trajectories of the letters, their execution and gestural control are dependent on both sensory feedback and the internal representation of the movement^5^, using a more mature scheme of the postural and segmental and articular organization of the writing arm^10,11^. With practice according to school levels, and therefore age, handwriting becomes more automatic and the control of movement becomes more and more proactive^10–12^. Thus, from the age of 9–10 years, the size of the strokes progressively increases and the gesture is less dependent on the visual control resulting from an internal representation of the movement. According to Schmidt's model, these internal representations are used by the cerebral cortex to determine a motor program^13,14^. The movements are then automatized, the letters are produced in an open loop, and visual feedback is only necessary to control the spatial arrangement of the letters in the word, the words on the line and the lines on the page^15^. The writing gesture is then sufficiently controlled to allow the correct adjustment of the letters’ size and their location in the writing space.

Few recent studies have analysed the influence of visual control on handwriting quality. The study by Chartrel and Vinter^16^ analyzed the spatial, temporal and kinematic organization of handwriting in children aged 8–10 years and adults under three visual conditions: with a normal vision, under an opaque open box that prevents to see the hand and the trace, or without any visual information. The results conclude that the absence of visual feedback (and thus visual control) in children results in decreased letter quality and increased strokes duration, dysfluence (discontinuity of movement), letter size, and pen pressure. The increase in letter size and pen pressure may be related to increased attention to kinesthetic feedback in the absence of visual feedback. Similar results are found in a recent study by Guilbert et al.^17^ involving elementary school students aged 7–11 years and adults. However, as far as we know, there are no studies on the correlations between visual control and postural and gestural organization of the child during drawing or handwriting. However, some studies have looked at the effect of lack of visual control on postural organization in anti-gravity. Several of those have shown that young children (up to 10 years old) have difficulties in resolving sensory conflicts and still have poor adaption abilities to sensory perturbations, such as visual feedback deprivation^18–20^. According to Berthoz et al.^21^, in the absence of visual control, postural righting and control abilities have a reflex function of vestibular origin. We can therefore hypothesize that, in the case of the child who draws or writes by hand, the suppression of vision could lead to kinesthetic compensation to control the graphomotor gesture.

The main hypotheses put forward to explain graphomotor disturbances assume a defect in motor programming or in motor execution that does not allow the automation of the strokes. Thus, Wann^22^ suggests a motor programming defect characterized by an alteration in the temporal organization of handwriting (dysfluence, high pause times) due to an excessive dependence on visual feedback. For Hamstra-Bletz and Blöte^23^, dysgraphia is a disturbance in the production of literacy partly due to a lack of fine motor control in the execution of motor programs. This hypothesis is also retained by Van Dorn and Keuss^24^ who found motor programming difficulties leading to an excessive use of visual information to control and execute the writing gesture. Benoit and Soppelsa^25^ assume the existence of two types of dysgraphia: spatial dysgraphia based on visual difficulties and motor dysgraphia for which kinaesthetic aspects must also be considered.

Neurologically, the Exner zone, located in an area of the left premotor cortex, is considered to contain the motor programs necessary for the production of letters^26^. In addition, studies in functional neuroimaging (fMRI) conclude to an involvement of large cortical areas at the frontal, temporal, parietal and occipital level and of the cerebellum^27,28^, and to differences in functional neuronal connections between white matter and gray matter in children with dysgraphia or dyslexia compared to typical children during a spelling judgment task^29^. The primary motor cortex, sensorimotor cortex, supplementary motor area (AMS), thalamus, and putamen would be involved in motor control, while the ventral pre-motor cortex and posterior/inferior temporal cortex would also involved in linguistic processes^30^.

In the present study, we compared the graphomotor gesture of typically developing children and children with handwriting disorders during a prescriptural task of copying a line of loops (previouly validated^10,11^) performed in two conditions: one with eyes openned, the other with eyes closed. We analyzed the effect of vision suppression both on the children’s postural and gestural organization and on the spatial, temporal and kinematic parameters of the loop drawings. The underlying hypothesis being that writing disorders could be partly explained by a deficit in the processing of sensory feedback during handwriting.

## Results

### Comparison of postural and gestural organization depending on the task (eyes open or eyes closed): typical group

#### Characteristics of the typical group

The population of children (n = 35) is composed of 26% girls (n = 9) and 74% boys (n = 26). There is no significant difference in the distribution of the children in our sample according to gender for the postural and gestural variables (MANOVA, p = 0.45). The characteristics of these children are presented in Table 1.

**Table 1:** Characteristics of children of the typical group for the postural and gestural parameters.

	Distribution by grade level					
	1st grade (n = 9)	2d grade (n = 8)	3th grade (n = 4)	4th grade (n = 4)	5th grade (n = 10)	Total (n = 35)
Age (months) M (SD)	79.11 (1.90)	89.25 (2.60)	100.00 (3.56)	113.00 (4.24)	123.10 (4.70)	100.26 (18.17)
Gender G(n)/B(n)	4/5	1/7	1/3	0/4	3/7	9/26

*M* mean, *SD* standard deviation, *n* number of children, *G* girls, *B* boys.

#### Postural and gestural organization in the typical group

The Table 2 presents the distribution (number of children) of the modalities of each of the postural and gestural variables studied depending to the experimental task.

**Table 2:** Repartition (n) of postural-gestural parameters eyes open vs eyes closed in the typical group.

	Variables	Modalities	Eyes openned (n = 35)	Eyes closed (n = 35)	*p*
Proximal gestural organization	Position of the head relative to the table	Close to the table	7	1	0.0004 ***
		Away from the table	28	34	
	Vertebral axis	Close to the table	15	3	0.003 **
		Away from the table	20	32	
	Shoulder elevation	Yes	21	10	0.02 *
		No	14	25	
	Elbow elevation	Yes	5	3	0.71
		No	30	32	
	Dynamic movement of elbow	Moving	33	27	0.09
		Static	2	8	
	Forearm elevation	Yes	7	7	1
		No	28	28	
	Dynamic movement of forearm	Lateral movement	27	26	1
		Rotation around the elbow	8	9	
Distal gestural organization	Wrist elevation	Yes	9	8	1
		No	26	27	
	Wrist rotation	Half supination	28	29	7.32e−05***
		Side slice	7	5	
		Pronation	0	1	
	Wrist in relation to the axis of the arm	Flexion	1	1	1
		In the axis	27	27	
		Extension	7	7	
	Dynamic movement of wrist	Static	22	23	0.82
		Flexion–extension	10	8	
		Hand rotation around the wrist	3	4	
	Pattern of pen grip	Classic tripod grip	18	18	1
		Non academic tripod grip	4	4	
		Quadripodic	13	13	
	Digital mobility	Flexion–extension	14	26	0.004**
		Static	21	9	
Gestural organization in relation to the material	Sheet tilt	Yes	15	18	0.63
		No	20	17	
	Position of the sheet	Vertebral axis	18	14	0.47
		Dominant right hemi-field	17	21	
	Position of the hand/line	On the line	26	22	0.57
		Below	8	12	
		Above	1	1	
	Fingers position on the pen	Classic	28	32	0.38
		Low	4	2	
		High	3	1	
	Pen tilt	Lying in the commissure between thumb and index	29	31	0.73
		Vertical	6	4	
	Control of gesture	Harmonious	14	19	0.22
		Hypercontrol	19	12	
		Precipitation	2	4	
	Type of gesture organization	Distal	26	27	1
		Proximal	9	8	
	Pressure on the pen	Balanced	21	22	1
		Hypertonic	14	13	
		Hypotonic	0	0	
	Oral-facial synkinesia	Yes	17	16	1
		No	18	19	

Significant values are in bold.

Levels of signification: *p < 0.05; **p < 0.01; ***p < 0.001.

Only 5 postural and gestural variables significantly differ according to the task (eyes openned or eyes closed). First, the “position of the head relative to the table”: the head is more frequently away from the table when drawing with the eyes closed. Secondly, the “position of the trunk (vertebral axis) in relation to the table”: the trunk is more frequently distant from the table when drawing with the eyes closed. Next, the “shoulder elevation”: the shoulders are more frequently relaxed downwards when drawing with the eyes closed. Then, the “wrist rotation”: the most academic position of the hand in semi-supination is more frequent when drawing with the eyes closed. Finally, the “digital mobility”: the fingers flexion–extension movements are more numerous when drawing with the eyes closed.

### Comparison of spatial–temporal and kinematic parameters depending on the task (eyes openned or eyes closed): typical group

#### Characteristics of the typical group

The population of children (n = 32) is composed of 31% girls (n = 10) and 69% boys (n = 22). There is no significant difference in the distribution of the children in our sample according to gender for the spatial–temporal and kinematic variables (MANOVA, p = 0.69). The characteristics of these children are presented in Table 3.

**Table 3:** Characteristics of children of the typical group for the spatial–temporal and kinematic parameters.

	Distribution by school grade level					
	1st (n = 9)	2d (n = 6)	3th (n = 4)	4th (n = 3)	5th (n = 10)	Total (n = 32)
Age (months) M (SD)	79.44 (2.19)	90.50 (2.17)	100.00 (3.56)	112.33 (5.03)	124.70 (4.88)	101.31 (19.02)
Gender G(n)/B(n)	4/5	1/5	1/3	1/2	3/7	10/22

*M* mean, *SD* standard deviation, *n* number of children, *G* girls, *B* boys.

#### Spatial–temporal and kinematic parameters of drawing in the typical group

The Table 4 presents the mean (standard deviation) of spatial, temporal and kinematic variables depending to the experimental task.

**Table 4:** Mean (± standard deviation) of spatial, temporal and kinematic variables eyes openned *vs* eyes closed in the typical group.

Spatial, temporal, kinematic variables	Eyes openned (n = 32) M (SD)	Eyes closed (n = 32) M (SD)	*p*
Number of strokes	2.88 (3.4)	1.62 (1.39)	0.29
Total drawing time (s)	21.59 (12.1)	21.34 (10.93)	0.90
Effective drawing time (s)	19.81 (10.33)	20.12 (9.35)	0.68
On-paper pauses times (s)	0.25 (1.02)	1.78 (2.2)	0.0002***
In-air pauses times (s)	0.81 (2.38)	1.09 (2.99)	0.93
Number of velocity peaks	0.66 (1.21)	1.19 (1.91)	0.21
Total drawing length (mm)	691.81 (142.01)	666.72 (143.92)	0.40
Average length per stroke (mm)	475.16 (251.04)	534.47 (202.66)	0.49
Average velocity (mm/s)	41.28 (14.98)	39 (16.2)	0.39
Maximum velocity (mm/s)	43.44 (13.86)	40.06 (15.85)	0.22
Drawing width (mm)	199.12 (5.98)	193.34 (9.73)	0.03*
Drawing height (mm)	21.94 (8.68)	27.41 (9.93)	0.02*
Number of loops	22.25 (7.39)	19.28 (6.17)	0.10
Degree of inclination of the line	− 1.44 (3.33)	0 (4.85)	0.36
Height of loops (mm)	8.94 (3.14)	9.5 (2.59)	0.26
Spacing between loops (mm)	9.69 (2.68)	10.97 (3.3)	0.18

Significant values are in bold.

Levels of signification: *p < 0.05; **p < 0.01; ***p < 0.001.

Only 3 spatial, temporal, or kinematic variables differed significantly by task (eyes openned or eyes closed): average on-paper pauses times increased when the child had eyes closed; average drawing width decreased when the child had eyes closed; average drawing height increased when the child had eyes closed.

### Comparison of postural and gestural organization depending on the task (eyes openned or eyes closed): handwriting disorders (HD) group

#### Characteristics of the HD group

The population of children (n = 35) is composed of 20% girls (n = 7) and 80% boys (n = 28).

There is no significant difference in the distribution of the children in our sample according to gender, nor for the postural and gestural variables (MANOVA, p = 0.97), nor for the spatial–temporal and kinematic variables (MANOVA, p = 0.75). The characteristics of these children are presented in Table 5.

**Table 5:** Characteristics of children of the handwriting disorders (HD) group.

	Distribution by grade level					
	1st grade (n = 9)	2d grade (n = 8)	3th grade (n = 4)	4th grade (n = 4)	5th grade (n = 10)	Total (n = 35)
Age (months) M (SD)	75.89 (3.44)	90.37 (5.50)	102.75 (2.99)	109.50 (2.65)	125.9 (4.33)	100.4 (19.99)
Gender G(n)/B(n)	3/6	0/8	1/3	0/4	3/7	7/28

*M* mean, *SD* standard deviation, *n* number of children, *G* girls, *B* boys.

#### Postural and gestural organization in the HD group

The Table 6 presents the distribution (number of children) of the modalities of each of the postural and gestural variables studied depending to the experimental task.

**Table 6:** Repartition (n) of postural-gestural parameters eyes openned *vs.* eyes closed in the handwriting disorders (HD) group.

	Variables	Modalities	Eyes openned (n = 35)	Eyes closed (n = 35)	*p*
Proximal gestural organization	Position of the head relative to the table	Close to the table	18	30	0.006**
		Away from the table	17	5	
	Vertebral axis	Close to the table	17	29	0.006**
		Away from the table	18	6	
	Shoulder elevation	Yes	16	8	0.02*
		No	19	27	
	Elbow elevation	Yes	10	6	0.39
		No	25	29	
	Dynamic movement of elbow	Moving	33	31	0.67
		Static	2	4	
	Forearm elevation	Yes	5	5	1
		No	30	30	
	Dynamic movement of forearm	Lateral movement	32	31	1
		Rotation around the elbow	3	4	
Distal gestural organization	Wrist elevation	Yes	13	12	1
		No	22	23	
	Wrist rotation	Half supination	25	26	0.96
		Side slice	9	8	
		Pronation	1	1	
	Wrist in relation to the axis of the arm	Flexion	3	3	0.96
		In the axis	22	23	
		Extension	10	9	
	Dynamic movement of wrist	Static	15	15	0.92
		Flexion–extension	17	16	
		Hand rotation around the wrist	3	4	
	Pattern of pen grip	Classic tripod grip	21	21	1
		Non academic tripod grip	4	4	
		Quadripodic	10	10	
	Digital mobility	Flexion–extension	15	18	0.65
		Static	20	17	
Gestural organization in relation to the material	Sheet tilt	Yes	19	20	1
		No	16	15	
	Position of the sheet	Vertebral axis	23	26	0.60
		Dominant right hemi-field	12	9	
	Position of the hand/line	On the line	22	23	0.59
		Below	10	11	
		Above	3	1	
	Fingers position on the pen	Classic	24	24	0.91
		Low	6	2	
		High	5	6	
	Pen tilt	Lying in the commissure between thumb and index	28	30	0.75
		Vertical	7	5	
	Control of gesture	Harmonious	6	12	0.26
		Hypercontrol	25	20	
		Precipitation	4	3	
	Type of gesture organization	Distal	15	21	0.23
		Proximal	20	14	
	Pressure on the pen	Balanced	13	17	0.62
		Hypertonic	21	17	
		Hypotonic	1	1	
	Oral-facial synkinesia	Yes	19	18	1
		No	16	17	

Significant values are in bold.

Levels of signification: *p < 0.05; **p < 0.01; ***p < 0.001.

Among children with handwriting disorders, only 3 postural and gestural variables differ significantly according to the task (eyes openned or eyes closed), which is less than among typically developing children (3 variables). First, the “position of the head in relation to the table”: the head is frequently closer to the table when drawing with the eyes closed (p < 0.01), unlike typically developing children who straighten their head when the eyes are closed. Second, the “position of the trunk (vertebral axis) in relation to the table”: the trunk is more frequently close to the table when drawing with eyes closed (p < 0.01), unlike typically developing children who move their trunk away from the table when their eyes are closed. Finally, the “shoulder elevation”: as in typically developing children, the shoulders are more frequently relaxed downward when drawing with eyes closed (p < 0.05).

### Comparison of spatial–temporal and kinematic parameters depending on the task (eyes openned or eyes closed): handwriting disorders (HD) group

The Table 7 presents the mean (standard deviation) of spatial, temporal and kinematic variables depending to the experimental task.

**Table 7:** Mean (± standard deviation) of spatial, temporal and kinematic variables eyes openned *vs* eyes closed in the handwriting disorders (HD) group.

Spatial, temporal, kinematic variables	Eyes openned M (SD)	Eyes closed M (SD)	*p*
Number of strokes	6.03 (6.94)	2.28 (2.1)	9.23e−04***
Total drawing time (s)	34.97 (21.38)	25.78 (11.79)	0.08
Effective drawing time (s)	28.09 (13.99)	23.47 (9.81)	0.17
On-paper pauses times (s)	0.47 (2.3)	2.06 (2.63)	8.45e−04***
In-air pauses times (s)	6.78 (9.95)	2.25 (4.85)	9.95e−04***
Number of velocity peaks	1.97 (2.43)	1.81 (2.92)	0.44
Total drawing length (mm)	768.53 (156.94)	741.66 (217.15)	0.45
Average length per stroke (mm)	296.28 (264.3)	504.53 (271.05)	0.002**
Average velocity (mm/s)	34.59 (19.27)	36.41 (17.54)	0.47
Maximum velocity (mm/s)	40.16 (19.32)	39.75 (19.12)	0.92
Drawing width (mm)	192.09 (14.24)	189.69 (12.13)	0.15
Drawing height (mm)	24.28 (7.24)	33.38 (12.38)	0.003**
Number of loops	26.72 (11.44)	20.31 (8.31)	0.02*
Degree of inclination of the line	− 0.5 (3.49)	− 2.34 (5.87)	0.25
Height of loops (mm)	9.62 (4.26)	10.66 (4.57)	0.33
Spacing between loops (mm)	8.81 (4.18)	10.91 (4.01)	0.02*

Significant values are in bold.

Levels of signification: *p < 0.05; **p < 0.01; ***p < 0.001.

**Figure 1:**
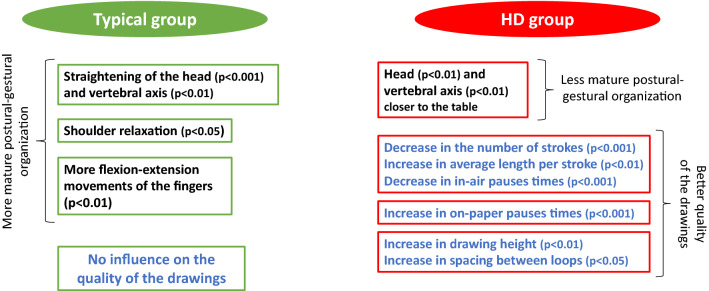
Significantly different variables between the two conditions (eyes openned vs eyes closed) in typical and HD groups (postural-gestural organization features in black; spatio-temporal and kinematic features in blue).

Among children with handwriting disorders, only 7 variables differed significantly by task (eyes openned or eyes closed, Fig. 1), which was more than in typically developing children (3 variables): the average on-paper pauses times increased when the child had eyes closed (p < 0.001); the average drawing width increased when the child had eyes closed (p < 0.01); the average number of strokes decreased when the child had eyes closed (p < 0.001), which means that he/she makes fewer in-air pauses; the average in-air pauses times decreases when the child has his/her eyes closed (p < 0.001); the average length per stroke increases when the child has his/her eyes closed (p < 0.01), which makes sense in relation to the decrease in the number of in-air pauses; the number of loops in the line decreases when the child has the eyes closed (p < 0.05); the spacing between loops increases when the child has the eyes closed (p < 0.05).

## Discussion

The children in our study were assessed using a simple, repetitive and early automated test (derived from a previously validated open-eye pregraphic task^10,11^) consisting in drawing a line of cycloid loops with eyes closed. Unlike handwriting, this task assesses the organization of the graphomotor gesture without requiring memorization, phonological integration, spelling, and long-term memory skills for the letter motor programs. Our results show, in typically developing children, a significant change in postural and gestural organization when tracing a line of cycloid loops with eyes closed. Indeed, when children close their eyes and move away from visual control, the postural and gestural organization becomes significantly more mature and relaxed: straightening the head and trunk, relaxing the shoulders, more academic position of the hand and more frequent flexion–extension movements of the fingers. This follows the evolution of postural and gestural norms highlighted from first to fifth grade in Vaivre-Douret et al.^10^ and Vaivre-Douret and Lopez^11^. On the other hand, closing the eyes had no significant influence on the spatial, temporal and kinematic organization of the loops. These results can be explained by data from the literature on the vestibular system. According to those, in the absence of visual control, there is a righting reflex of vestibular origin that precedes the initiation of proprioceptive reflexes^21^. It is therefore not surprising that in typically developing children, eye closure is associated with head and trunk straightening, perhaps in an effort to control their posture better. Our results showing the lack of influence of vision suppression on the temporal and kinematic parameters of drawing in our study are congruent with Smyth and Silvers study^31^, who conclude that there is no effect of the presence or absence of vision on the average times taken to write. However, they did not use a pre-scriptural task but a writing test on adults in a so-called "blind" condition, without using vision. They observed that the overall spatial arrangement and orientation of words were affected by the absence of visual feedback. On the other hand, the shape of words and the legibility of letters were a little degraded. This spatial invariance, maintained despite variations in the performance context, would be a typical and remarkable feature of handwriting^32^. Thus, our results on the spatial, temporal and kinematic organization of loops are congruent with the principle of motor equivalence, which attests of personal characteristics of handwriting stability, despite changes in effectors with which the letter is formed. Thus, we can assume the good integration of the internal representation of loop trajectories in typically developing children, who perform proactive control of these pre-scriptural traces (also called open-loop control). Neuroimaging studies have shown an absence of activation of the basal ganglia during open-loop control, whereas they are highly activated (as well as the left anterior putamen) during non-automated closed-loop control^33^. These results could support the hypothesis of a lack of involvement of the cortico-striatal pathway when the child has developed a good internal representation of the drawing/letter and is at a late stage of learning consolidation. Since the Exner area is considered to contain the motor programs necessary for letter production^26^, this area could also be implicated in handwriting disorders in ours ample of children.

However, our results are very different among children with handwriting disorders. Indeed, for them, tracing the line of loops blindly results in significantly poorer postural control, manifested by bringing the head and trunk closer to the table and by a statistical tendency for more mature inter-segmental movement gestures. Van Dorn and Keuss^24^ conclude that suppression of visual information has a beneficial effect for poor writers on the movement quality when finger movements are used. On the other hand, the authors find a negative effect of this suppression on the production of wrist movements. If the postural straightening in typically developing children can be explained by a vestibular-based straightening reflex, it can be speculated that the poorer postural quality in children with handwriting disorders when the eyes are closed may be due to proprioceptive deficits in these children, many of whom (52%) have a kinaesthetic memory disorder^34^. This hypothesis is reinforced by the results of several studies which have shown the influence, on the one hand of kinesthetic perception^35,36^ and, on the other hand of postural control^37–40^ on handwriting development.

At the same time, in the blind loop test, children in our sample with handwriting disorders draw significantly smoother (shorter in-air pauses [p = 9.95e−04]); make larger spacing between loops [p = 0.02]; make larger loops (increased drawing line height [p = 0.003] and average length per stroke [p = 0.002]).

These results are in agreement with those of Chartrel and Vinter^41^ that children tend to maximize kinaesthetic information by increasing letter size when writing in the absence of visual feedback. The improvement in the spatial, temporal and kinematic organization of the drawing when the children detach from visual control could be explained by a disruptive effect of visual control on the spatial, temporal and kinematic parameters of the drawings among these children, many of whom have visuospatial disabilities (78% of them have difficulties with visuospatial graphomotor coordination^34^). These results are corroborated by several studies which have shown the link between visuomotor integration capacities (evaluated by the VMI test) and handwriting skills^42–44^. Moreover, interestingly, in patients with Parkinson's disease, several authors^45,46^ have validated the hypothesis that impaired utilization of sensory feedback may retard the effective learning of motor programs. Teulings et al.^46^ have shown in particular that patients with Parkinson's disease do not adapt their visuomotor map in response to a distorted visual feedback of handwriting but rely constantly on the visible trace feedback during the ongoing movement.

Thus, in our sample, it can be assumed that children with handwriting disorders have difficulties in achieving proactive control of loop drawings, which remains dependant on visual and kinaesthetic feedbaks, themselves insufficiently efficient. This would explain the overactivation of the visual system and of parietal and cerebellar cerebral brain regions by the poor writers identified in studies^47^. In fact, closed loop control (feedback control) would lead to a strong activation of the basal ganglia and the putamen. Thus, we can assume that the learning of pre-graphic patterns in children with handwriting disorders would remain dependent on the cortico-cerebellar pathway, which is only active at the beginning of learning in typically developing children^32^. It is possible that children with handwriting disorders need to overactivate parietal and cerebellar regions as well to compensate for difficulties in timing, which are notable in our sample of children. These hypotheses are supported by knowledge about the proprioceptive role of vision^48^ which participates in tonic and postural regulation by informing the brain, through retinal information, about the position and movement of the body in space. Peripheral vision is also involved in postural control by informing the brain about the orientation of the individual in relation to the environment^21^.

Our experiment would deserve to be enriched by cross-sectional analyzes, in particular visual-motor coordination, to complement our conclusions that the development of the graphomotor gesture involves kinaesthetic, visual and motor sensory activities. Moreover, our experiment would deserve to be replicated on a larger sample of children. In spite of this, this current research is an interesting contribution liable to enhance clinical practices, useful in clinical decision-making processes for handwriting disorders remediation. Indeed, these results underline the interest, on children with handwriting disorders, of proposing letter learning techniques aimed at modifying the perception of handwriting in real time. For example, methods aiming at reinforcing the kinaesthetic feedbacks, in particular by making the child feel the correct kinematics of the movement or by making him/her go through haptic learning allowing a better memorization of the pre-graphic patterns seem interesting^49–51^. It would also be interesting to explore the effect of suppressing visual feedback on letter learning on children with handwriting disorders, as has been done on typical adults^52^. The goal would be to reduce the cognitive overload caused by the dual task of processing visual information, which can be complex for children with handwriting disorders and visuospatial difficulties. The child, by privileging kinaesthetic learning allowing the visualization of a mental image of the letter, could then better integrate the correct trajectory of the letter despite possible visuospatial difficulties. Clinical practices and remediation of handwriting disorders would therefore benefit from techniques that promote multisensory learning of letters to compensate for the potential difficulties in integrating sensory feedback encountered by these children and to compensate their potential visual difficulties.

## Materials and methods

### Participants

Data from a sample of 35 children with handwriting disorders (HD group) aged 6 years 2 months to 11 years 11 months (mean 8.40 SD 1.70) and 35 matched typical children (typical group) were collected from elementary school (grades 1 to 5) in Paris, France. Children were excluded from the study if they had prematurity (birth < 37 WA), sensory, visual, neurological or genetic disorders, dyslexia and severe language disorder, ADHD (according to the DSM-5 criteria), autism spectrum disorder, psychopathology, or motor disorder caused by injury or accident. None of them had repeated or skipped a grade. The institutional research ethics committee of Paris Descartes University approved the study procedures (CER·2018-72) conducted in accordance with the Declaration of Helsinki. All parents and children provided written informed consent.

### Design and measures

Handwriting disorders among the HD group were detected by the teachers and objectified by an analysis of their school notebooks by an experienced psychomotor therapist. In order to assess their handwriting level, all children were submitted to a standardized French handwriting assessment, the BHK scale^53^ adapted from the Concise Evaluation Scale for children’s handwriting^54^. Children were included in the typical group if their BHK score was within the average for their age.

### Experimental handwriting assessments

**Figure 2:**
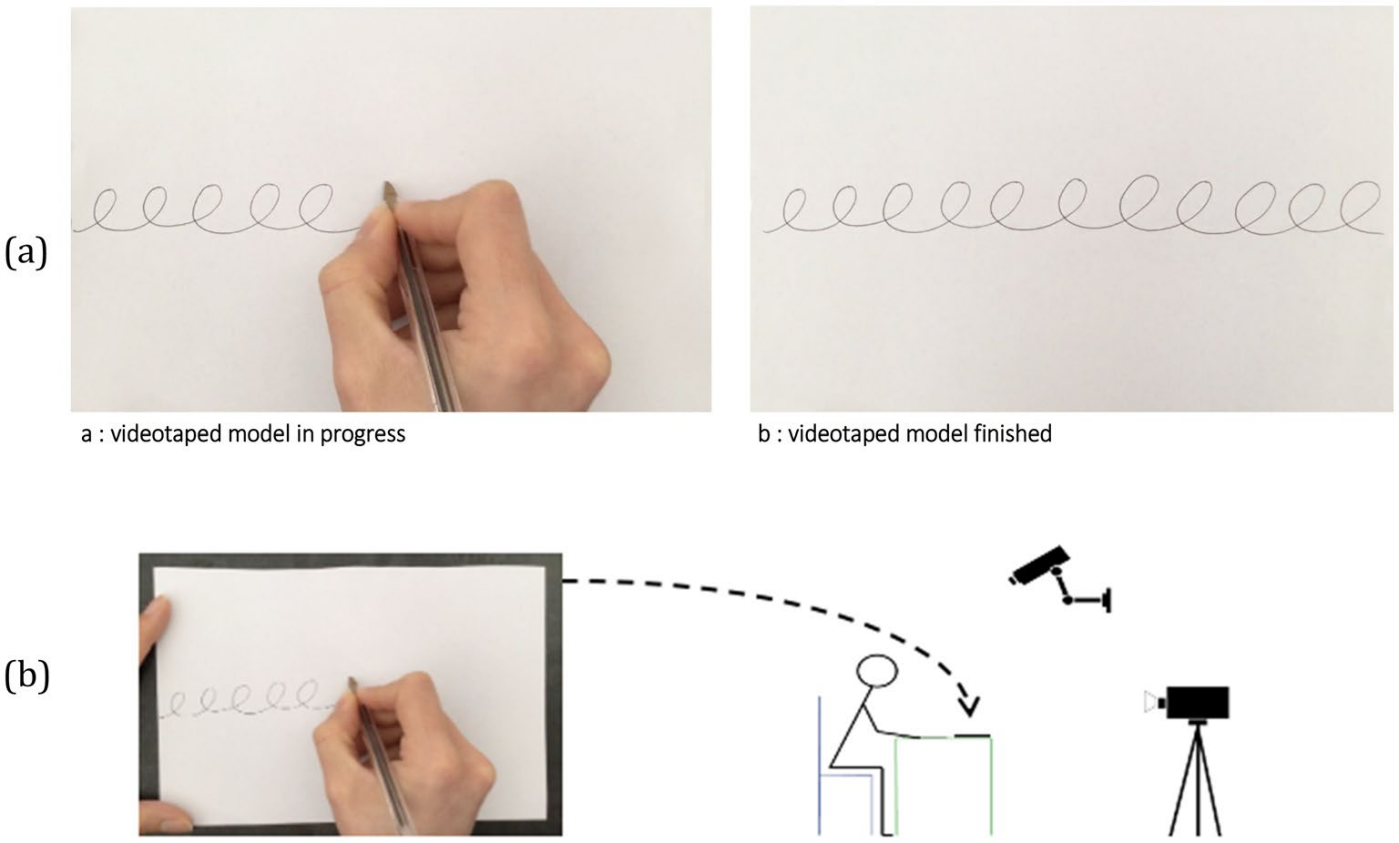
(**a**) Setting: iPad tablet placed in front of the child presenting the model of the copy of a line of cycloid loops. The child was video-recorded drawing the copy of the line of cycloid loops with a digital pen on a paper sheet put on the table. (**b**) Extract from the videotaped model presented to the child on the iPad tablet for the model of the copy of a line of cycloid loops. Source: Vaivre-Douret et al. (2021), https://doi.org/10.1038/s41598-020-79315-w.

All children completed a pre-scriptural task, which consisted in copying a previously validated cycloid loop line test to assess developmental levels of handwriting^10,11^ (see Fig. 2). Data on postural organization and inter-segmental coordination of the writing arm (video recording with two cameras followed by 2D reconstruction) and spatio-temporal and kinematic measures (using a digital pen) were systematically collected. The child was placed in the most ecological environment possible, on a chair in front of a table, feet flat on the ground, forearms flat on the table without raising the shoulders. A half sheet of A4 paper was positioned widthwise, aligned straight in front of the child, who was free to move it. The loop pattern, identical for all children, was presented on an iPad tablet placed in front of the child.

### Postural and gestural parameters

The video recordings enabled to analyze the parameters concerning the proximal (head, trunk axis, shoulder, elbow and forearm) and distal (wrist and fingers) segments and joints in the coordinated gestural organization of the drawing process, as well as variables reflecting the organization of positioning in relation to the material (sheet, drawing line, pen). In addition, observational clinical variables related to the semiology of the motor characteristics of the gesture (control, pressure, synkinesis) were considered (see Vaivre-Douret et al.^10^ and Vaivre-Douret and Lopez^11^).

### Spatial–temporal and kinematic measures

**Figure 3:**
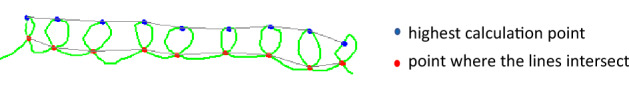
Extracted from the handwriting analysis device (Elian Research software, Version 4.2, http://www.seldage.com) for the copy of a line of cycloid loops. Each point forming the line of loops and the highest point of the loop, the intersection point are recorded for the calculation of the following features: total drawing length, average length per stroke, drawing width, drawing height, degree of inclination of the line, height of the loops, spacing between loops, number of loops, number of strokes, total drawing time, effective drawing time, number of on-paper pauses, number of in-air pauses, number of velocity peaks, average and maximum velocity.

The recording of spatial–temporal and kinematic parameters was conducted for all the HD group and for 32 children of the typical group (because of a data logging problem) using an independent Anoto electronic digital pen, connected after the assessment to a handwriting analysis device (Elian Research software, Fig. 3). The unlined paper sheet comprised a single set of dots printed in ‘watermark’ mode (Anoto). The pen was presented to the child vertically on the sheet so as not to influence the child’s choice of the hand with which to write.

The measures collected with the electronic pen were the following:

- Spatial parameters: total drawing length (length of all strokes), average length per stroke (length measured by stroke, a stroke corresponding to a continuous lines, without lifting the pen from the sheet of paper), drawing width (difference between the rightmost point of the cycloid loops line and the leftmost point of the line), drawing height (difference between the highest point of the cycloid loops line and the lowest point), degree of inclination of the line (average inclination of the loop line from the horizontal), height of the loops (average height of each loop), spacing between loops (average of the spaces between each of the loops in the line), number of loops (number of loops drawn in the entire line).
- Temporal parameters: number of strokes (number of continuous lines, without lifting the pen from the sheet of paper), total drawing time (total time taken by the child to complete the loop line, including both the times the pen traces on the sheet, and the paused times when the pen does not make any traces), effective drawing time (tracing time during which the pen is in motion and in contact with the sheet), number of on-paper pauses (pauses when the pen is no longer drawing and during which it is in contact with the sheet), number of in-air pauses (pauses when the pen is no longer drawing and when it is lifted from the sheet).
- Kinematic parameters: number of velocity peaks (moments of acceleration of the drawing before deceleration), average and maximum velocity (ratio of the average/maximum total plot time to the average/maximum total plot length).

### Statistical analyses

The statistical analyses were carried out on R software (version 3.5.3). The degree of significance retained for all assignments was set at 0.05. In order to compare the postural and gestural organization of children between the two pre-scriptural task, one with the eyes open and the other with the eyes closed, a statistical test of χ^2^ was carried out after obtaining contingency tables for each of the variables. In order to compare the spatial–temporal and kinematic parameters of the drawing between the two pre-scriptural task, a statistical test of Wilcoxon was carried out.
